# Antimalarial Activity of Methoxylated Flavonoid Fraction (Cirsimaritin-Dominated) from *Artemisia kopetdaghensis* in *Plasmodium berghei*-Infected Mice with in silico Analysis

**DOI:** 10.5812/ijpr-167100

**Published:** 2026-04-26

**Authors:** Roya Amirian, Seyed Hossein Hejazi, Naser Ghoddosi, Hamed Fouladseresht, Morteza Sadeghi, Seyed Mahmoud Mousavi, Zeinab Yazdiniapour, Maryam Fattahian, Mustafa Ghanadian

**Affiliations:** 1 Department of Parasitology and Mycology, Isfahan Pharmaceutical Sciences Research Center, School of Pharmacy, Isfahan University of Medical Sciences, Isfahan, Iran; 2 Department of Parasitology and Mycology, School of Medicine, Isfahan University of Medical Sciences, Isfahan, Iran; 3 Department of Parasitology and Mycology, Skin Disease and Leishmaniasis Research Center, School of Medicine, Isfahan University of Medical Sciences, Isfahan, Iran; 4 Department of Immunology, School of Medicine, Isfahan University of Medical Sciences, Isfahan, Iran; 5 Department of Biochemistry, Sanandaj Branch, Islamic Azad University, Sanandaj, Iran; 6 Department of Pharmacognosy, Isfahan Pharmaceutical Sciences Research Center, School of Pharmacy, Isfahan University of Medical Sciences, Isfahan, Iran

**Keywords:** Malaria, Methoxylated Flavonoid Fraction, *Artemisia kopetdaghensis*, Molecular Docking, Cirsimaritin

## Abstract

**Background:**

Building on our previous investigation of the total semipolar extract (SPE) of *Artemisia kopetdaghensis* in *Plasmodium berghei*-infected mice, the present study focuses specifically on the methoxylated flavonoid fraction (MFF). Flavonoids are increasingly recognized for their ability to suppress parasite growth and modulate host immunity. To clarify their role, we isolated and characterized the major flavonoid constituents of this plant and evaluated their antimalarial potential, both alone and in combination with chloroquine, in a mouse model.

**Methods:**

Aerial parts of *A. kopetdaghensis* were extracted using a chloroform:acetone (2:1) solvent mixture. The extract was then fractionated by column chromatography. Based primarily on ^1H-NMR spectra, the MFF was selected and further purified by preparative HPLC. Isolated compounds were identified by 1D and 2D NMR and mass spectrometry. In the in vivo antimalarial study, thirty-six infected female Balb/c mice were treated with MFF and evaluated for key parameters. Docking and molecular interaction studies were conducted using AutoDock v4.2.6 software to examine the interactions of constituents with cytokine protein targets: 1D9C [interferon-gamma (IFN-γ)], 1B6C (TGF-β), 1BBN [(interleukin-4 (IL-4)], and 4HR9 (IL-17), separately.

**Results:**

Phytochemical analysis of the MFF by HPLC revealed three flavones: 6-methoxytricin (20%), cirsilineol (10%), and cirsimaritin (70%), identified for the first time in *A. kopetdaghensis*, with cirsimaritin as the dominant constituent. In vivo, MFF treatment significantly reduced parasitemia and enhanced parasite suppression in *P. berghei*-infected mice. Cytokine profiling demonstrated suppression of TGF-β and IL-4 followed by their recovery, together with marked elevations of IFN-γ and IL-17, indicating balanced modulation between pro-inflammatory and regulatory responses. These immunological findings were corroborated by molecular docking analyses, which confirmed binding interactions of cirsimaritin with cytokine receptor targets, providing mechanistic support for its immunomodulatory activity.

**Conclusions:**

This work extends our previous study on the SPE by moving from extract-level observations to constituent-specific insights. By combining phytochemical isolation with in silico receptor interaction analysis, we demonstrate that methoxylated flavonoids, particularly cirsimaritin, are key modulators of host immunity and promising candidates for further development as adjuncts or leads in antimalarial therapy.

## 1. Background

*Artemisia* species are currently being used to discover new medicines against malaria, most notably artemisinin, a sesquiterpene lactone obtained from *Artemisia annua* (1). Flavonoids are among the other secondary metabolites with significant bioactive effects (2). These compounds typically exhibit anti-inflammatory properties and act as immune system boosters (3). Most are potent antioxidants due to their chelating properties, which prevent the oxidation of metal ions such as iron and copper. Antimalarial effects, primarily demonstrated through in vitro assays, have been reported for *A. annua*, *A. turcomanica*, and other species (4).

Malaria infection triggers a complex host immune response in which cytokines play a dual role. Interferon-gamma (IFN-γ) activates macrophages to enhance the phagocytosis of parasitized erythrocytes and promotes parasite killing through nitric oxide production. Conversely, interleukin-4 (IL-4) drives Th2-type responses, promoting antibody production and B-cell differentiation. The IFN-γ/IL-4 ratio, therefore, serves as a useful indicator of Th1/Th2 balance during malaria infection (5). Interleukin-17 promotes neutrophil recruitment and enhances pro-inflammatory responses at infection sites, whereas transforming growth factor-beta (TGF-β) suppresses macrophage activation, inhibits pro-inflammatory cytokine production, and promotes regulatory T-cell responses. The balance between IL-17 (pro-inflammatory) and TGF-β (regulatory) reflects the Th17/Treg axis (6).

Previously, the antimalarial activity of the semipolar total extract of *Artemisia kopetdaghensis* (Syn: *Seriphidium kopetdaghense*) in a *P. berghei*-infected mouse model was reported (7). The terpenoid fraction of this plant was also analyzed, yielding several eudesmanolide sesquiterpenoids with cytotoxic properties against cancer cells (8, 9). Unlike earlier studies that focused on terpenoid constituents and the general antimalarial properties of *A. kopetdaghensis*, the present work identifies its methoxylated flavonoid fraction (MFF), with cirsimaritin as the dominant compound.

## 2. Objective

 It investigates the antimalarial efficacy of MFF in infected mice, with further insights into its immunomodulatory potential through serum cytokine analysis (IFN-γ, IL-4, IL-17, and TGF-β), and confirms the cytokine results by in silico studies to elucidate possible cytokine target protein interactions.

## 3. Methods

### 3.1. Chemicals

All chemicals, reagents, and solvents used in this study, including chloroform, acetone, methanol, ethyl acetate, hexane, trifluoroacetic acid, 2-aminoethyldiphenylborinate, and silica gel (mesh size 60–120) for column chromatography, were purchased from Pars-Chemie (Iran), Merck (Germany), or Sigma-Aldrich (USA), unless otherwise stated. HPLC-grade solvents were used for chromatographic separation on a Shimadzu C-18 end-capped preparative column (Japan). In animal studies, the method described by the same authors (7) was employed, and its description partly reproduces their wording.

### 3.2. Plant Material and Extraction

Aerial parts of *A. kopetdaghens* is were collected from the Bojnord region (North Khorasan, Iran) at the end of summer, specifically at Latitude: 36°38′20″ N and Longitude: 59°52′23″ E. The plant material was authenticated by a botanist using a voucher specimen (SAM-4005), which was deposited at the Samsam-Shariat Herbarium, School of Pharmacy, Isfahan University of Medical Sciences.

#### 3.2.1. Extraction

Air-dried aerial parts of *A. kopetdaghens* (9 kg) were coarsely ground and subjected to sequential maceration at room temperature with a dichloromethane–acetone (CH₂Cl₂–Me₂CO; 2:1 v/v) solvent mixture (3 × 40 L). Each maceration step was performed over a period of one week. The combined extracts were filtered and concentrated under reduced pressure using a rotary evaporator to yield 600 g of a dark green crude extract.

#### 3.2.2. Defatting and Pigment Removal

The crude extract was subjected to a vacuum liquid chromatography (VLC) system packed with reversed-phase silica gel (C18, 70 - 230 mesh) for initial cleanup. Unlike normal-phase chromatography, the non-polar stationary phase in reversed-phase VLC retains lipophilic compounds such as fats, waxes, and pigments. Accordingly, the column was eluted with an H₂O-MeOH mixture (30:70 v/v, 10 L), which selectively washed out semi-polar compounds, including methoxylated flavonoids, while non-polar constituents remained adsorbed onto the stationary phase. The remaining fats and non-polar constituents were subsequently eluted using pure methanol. The target semi-polar fraction was then concentrated to afford a defatted extract (186 g) (9).

#### 3.2.3. Column Chromatography

The defatted extract was fractionated by open-column chromatography (CC) on normal-phase silica gel (70 - 230 mesh, 1500 g). A gradient elution system of increasing polarity was employed, starting with hexane:ethyl acetate (Hex:EtOAc; 97:3) and gradually increasing the proportion of ethyl acetate to a final ratio of Hex:EtOAc (40:60), followed by the addition of methanol with a gradual increase from 1 to 30% (7).

#### 3.2.4. Fraction Monitoring and Selection

The column elution was monitored by thin-layer chromatography (TLC) using two distinct spray reagents for targeted detection. Fractions were primarily screened with vanillin-sulfuric acid (1% vanillin in 5% H_2_SO_4_) to visualize terpenoids. They were subsequently sprayed with a natural product reagent (2-aminoethyldiphenylborinate, 1% in MeOH) for the specific detection of flavonoids. Based on this dual-reagent analysis, Fractions 6 (3 g) eluted with hexane:ethyl acetate (60:40) and 7 (5 g) eluted with hexane:ethyl acetate (60:40) showed a strong yellowish fluorescence under the natural product reagent, indicating a high flavonoid content. Fractions displaying this strong fluorogenic response were subsequently subjected to preliminary ¹H-NMR spectroscopy. The presence of intense singlet resonances in the δH 3.5 - 4.2 ppm range, characteristic of methoxy (-OCH_3_) groups, alongside aromatic proton signals for flavones between δH 6.0 - 8.0 ppm, could be considered indicative of methoxylated flavones (10). Preliminary ¹H-NMR analysis of the combined fractions confirmed this, showing a predominance of signals characteristic of methoxylated flavones, along with minor resonances suggestive of sesquiterpene lactones.

#### 3.2.5. Precipitation and Flavonoid Separation

To separate these two classes of compounds, the combined material from Fr.6 and Fr.7 was subjected to two successive methanol precipitation steps. This process yielded a methanol-insoluble precipitate and a methanol-soluble filtrate. The resulting precipitate was collected, labeled as MFF, and set aside for the detailed isolation and identification of flavonoid constituents. The filtered methanol-soluble fraction, which contained sesquiterpene lactones, was processed separately and reported elsewhere (9).

#### 3.2.6. Final Purification

**Figure 1.**
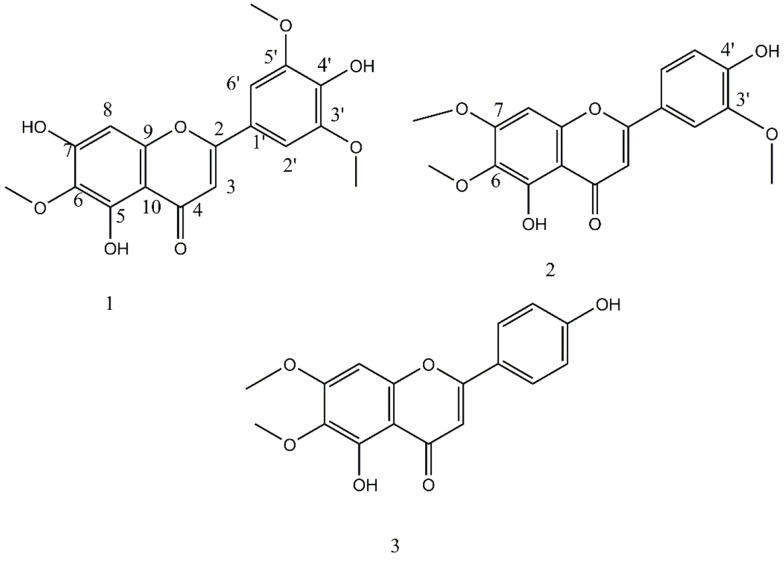
Metoxylated flavonoids of *Artemisia kopetdaghensis*

Methoxylated flavonoid fraction was further purified by high-performance liquid chromatography (HPLC) using a Waters semi-preparative system equipped with a Shimpack C-18 end-capped column (dimensions: 250 × 20 mm, particle size: 5 µm). The analysis was conducted at room temperature with UV detection at 250 nm. A stepwise gradient elution was applied using methanol, water, and trifluoroacetic acid (TFA): Fr.1: 65:35:0.1, 120 mL; Fr.2: 70:30:0.1, 120 mL; Fr.3: 75:25:0.1, 120 mL; Fr.4: 80:20:0.1, 120 mL; Fr.5: 85:15:0.1, 120 mL; and Fr.6: 90:10:0.1, 120 mL. The flow rate of the mobile phase was 3 mL/min, and the samples were collected in 10 mL fractions. The total run time per sample was approximately 3.3 min. Fractions 2 and 4 of the HPLC analysis yielded three peaks corresponding to compounds 1, 2, and 3, respectively, which were isolated in their pure states and subjected to further structural elucidation using NMR and MS. Their retention times were recorded at 79, 118, and 128 min, respectively (Figure 1). 

### 3.3. Spectral Data of Methoxylated Flavonoid Fraction Composition

#### 3.3.1. 6-Methoxytricin

Pale yellow powder. ^1H NMR (400 MHz, DMSO-d6) δ 3.75 (6H, s, 6-OMe), 3.88 (3H, s, 3′,5′-OMe), 6.70 (1H, s, H-8), 6.98 (1H, s, H-3), 7.32 (2H, s, H-3′,5′), 9.32 (1H, br s, 4′-OH), 10.77 (1H, br s, 7-OH), 13.08 (1H, br s, 5-OH). ^13C NMR (101 MHz, DMSO-d6) δ 56.82 (3′-OMe, 5′-OMe), 60.41 (6-OMe), 94.93 (C8), 103.60 (C3), 104.55 (C10), 104.80 (C2′,6′), 120.89 (C1′), 131.79 (C6), 140.29 (C4′), 148.65 (C3′,5′), 152.86 (C5), 153.17 (C9), 157.76 (C7), 164.19 (C2), 182.66 (C4). Negative ESI-MS at 359.3 m/z [M–H]⁻.

#### 3.3.2. Cirsilineol

Pale yellow powder. ^1H NMR (400 MHz, DMSO-d6) δ 3.73 (3H, s, 7-OMe), 3.90 (3H, s, 3′-OMe), 3.92 (3H, s, 6-OMe), 6.93–6.98 (3H, overlapped, H-8, H-3, H-5′), 7.58 (1H, d, J = 2.2 Hz, H-2′), 7.60 (1H, dd, J = 8.3, 2.2 Hz, H-6′), 10.06 (1H, br s, 4′-OH), 12.94 (1H, br s, 5-OH). ^13C NMR (101 MHz, DMSO-d6) δ 56.45 (3′-OMe), 56.90 (7-OMe), 60.48 (6-OMe), 92.07 (C8), 103.46 (C3), 105.52 (C10), 110.63 (C2′), 116.25 (C5′), 120.90 (C6′), 121.84 (C1′), 132.30 (C6), 148.50 (C3′), 151.33 (C4′), 152.51 (C5), 153.08 (C9), 159.07 (C7), 164.52 (C2), 182.66 (C4). Negative ESI-MS at 343.3 m/z [M–H]⁻.

#### 3.3.3. Cirsimaritin

Pale yellow powder. ^1H NMR (400 MHz, DMSO-d6) δ 3.74 (3H, s, 7-OMe), 3.93 (3H, s, 6-OMe), 6.82 (1H, s, H-8), 6.93–6.95 (3H, overlapped, H-3, H-3′, H-5′), 7.97 (2H, d, J = 8.6 Hz, H-2′,6′), 10.39 (1H, br s, 4′-OH), 12.91 (1H, br s, 5-OH). ^13C NMR (101 MHz, DMSO-d6) δ 56.64 (7-OMe), 60.25 (6-OMe), 91.78 (C8), 102.80 (C3), 105.22 (C10), 116.19 (C3′,5′), 121.21 (C1′), 128.75 (C2′,6′), 132.02 (C6), 152.21 (C5), 152.82 (C9), 158.81 (C7), 161.50 (C4′), 164.30 (C2), 182.43 (C4). Negative ESI-MS at 313.3 m/z [M–H]⁻.

### 3.4. In-vivo Antimalarial Study

The animal model and infection procedure were conducted as previously described (7). Briefly, in the 5-day treatment protocol, female Balb/c mice (6 - 8 weeks old) were inoculated intraperitoneally with *Plasmodium berghei*-infected erythrocytes. Treatment groups received intraperitoneal injections of MFF at 50, 100, or 150 mg/kg for three consecutive days, starting 48 hours post-infection. A combination group received MFF (100 mg/kg) plus chloroquine (10 mg/kg). Chloroquine alone (10 mg/kg) served as the positive control, while the vehicle (DMSO/Tween 80 in PBS) served as the negative control. Parasitemia was monitored by Giemsa-stained blood smears, and survival was recorded over 29 days. Parasite suppression was calculated relative to the negative control group.

### 3.5. Immunological Responses

To evaluate the role of cytokines in treatment prognosis, blood samples were collected from mice on days 0, 2, 4, and 7 post-infection. Sampling was performed from the orbital sinus under ketamine/xylazine anesthesia using heparinized capillary tubes. Following a 120-minute incubation, samples were centrifuged at 3000 rpm for 15 min, and the resulting serum was stored at -20 °C until analysis. Levels of IFN-γ, IL-4, interleukin-17 (IL-17), and transforming growth factor-β (TGF-β) were quantified using ELISA kits (R&D Systems, PBL Biomedical Laboratories, NJ, USA) as previously described (7, 11).

### 3.6. Molecular Docking Analyses

To confirm the cytokine results, docking and molecular interaction studies were conducted using AutoDock v4.2.6 software to examine the interactions of cirsimaritin with its cytokine protein targets: 1D9C (IFN-γ), 1B6C (TGF-β), 1BBN (IL-4), and 4HR9 (IL-17), separately. Initially, water molecules were removed from the receptor, and polar hydrogens and charge combinations, such as Coulombic and Gasteiger charges, were added and optimized. A grid box with dimensions of 70 × 80 × 75 Å and a spacing of 0.348 Å was defined around the active site residues, and a Lamarckian genetic algorithm (GA) was used to generate the binding outputs. The conformation with the lowest docking energy was selected for further analysis. The final best conformations were then visualized in 2D to illustrate interactions between ligands and receptor residues using Discovery Studio (DS 2017 R2 client).

### 3.7. Statistical Analysis

Statistical analysis was performed using SPSS (version 22) and one-way ANOVA, followed by Tukey’s post-hoc test. A P-value < 0.05 was considered indicative of a significant difference. The results are presented as the mean ± standard deviation (SD).

## 4. Results

### 4.1. Phytochemical Analysis

Purification of the MFF resulted in three methoxylated flavonoids (1-3). Compound 1 was isolated as a pale-yellow powder, with a negative ion at m/z 359.3 [M–H]⁻. ^13C NMR and DEPT spectra (DMSO-d6) revealed 18 distinct carbon signals characteristic of a flavone skeleton. These included a carbonyl resonance at δC 182.66 (C-4), eight oxygenated aromatic carbons, and two quaternary aromatic carbons at δC 104.55 (C-10) and 120.89 (C-1′). Four aromatic methine carbons were observed at δC 94.93 (C-8), 103.60 (C-3), and 104.80 (C-2′,6′), the latter accounting for two equivalent carbons. Additionally, three methoxy groups were identified at δC 60.41 (6-OMe) and 56.82 (3′-OMe and 5′-OMe).

The ^1H NMR spectrum (DMSO-d6) exhibited a chelated hydroxyl proton at δH 13.08 (1H, br s), attributed to the 5-OH group, while two additional hydroxyl signals were observed at δH 10.77 (1H, br s, 7-OH) and 9.32 (1H, br s, 4′-OH). Three methoxy groups were evident from singlets at δH 3.88 (6H, s, 3′-OMe and 5′-OMe) and 3.75 (3H, s, 6-OMe). The aromatic region displayed signals indicative of a symmetric B-ring, with a two-proton singlet at δH 7.32 (2H, s, H-2′/6′). Two additional one-proton singlets were observed at δH 6.98 (1H, s) and 6.70 (1H, s), assigned to H-3 and H-8, respectively, confirming the substitution pattern of the A-ring. Based on reference data reported by Hell et al., compound 1 was identified as 3′,5′,6-trimethoxy-5,7,4′-trihydroxyflavone, commonly known as 6-methoxytricin (12).

Compound 2 (4 mg) revealed a ^13C NMR spectrum with 18 carbon signals, including three methoxy groups at δC 56.45 (3′-OMe), 56.90 (7-OMe), and 60.48 (6-OMe), as well as 14 olefinic carbons and one carbonyl carbon at δC 182.71 ppm. Based on NMR data, it was identified as 3′,6,7-trimethoxy-5,4′-dihydroxyflavone, known as cirsilineol (13).

Compound 3 (32 mg) was similar to compound 2, except for the ^13C and ^1H NMR data of ring B. The ^1H NMR spectrum of ring B exhibited two ortho-coupled olefinic methine protons as AA′BB′ doublets with a coupling constant of 8.6 Hz at δC 116.19 (δH 6.95) and 128.75 (δH 7.97) ppm, each integrating for two protons and assigned to H-3′,5′ and H-2′,6′ in ring B, respectively. Additionally, the spectrum showed two methoxy groups at δH 3.74 (7-OMe) and 3.93 (6-OMe), respectively. Therefore, according to the literature and in agreement with its negative ESI-MS at 313.3 m/z [M–H]⁻, it was identified as 6,7-dimethoxy-5,4′-dihydroxyflavone, also known as cirsimaritin (14).

### 4.2. In-vivo Antimalarial Activity

**Figure 2.**
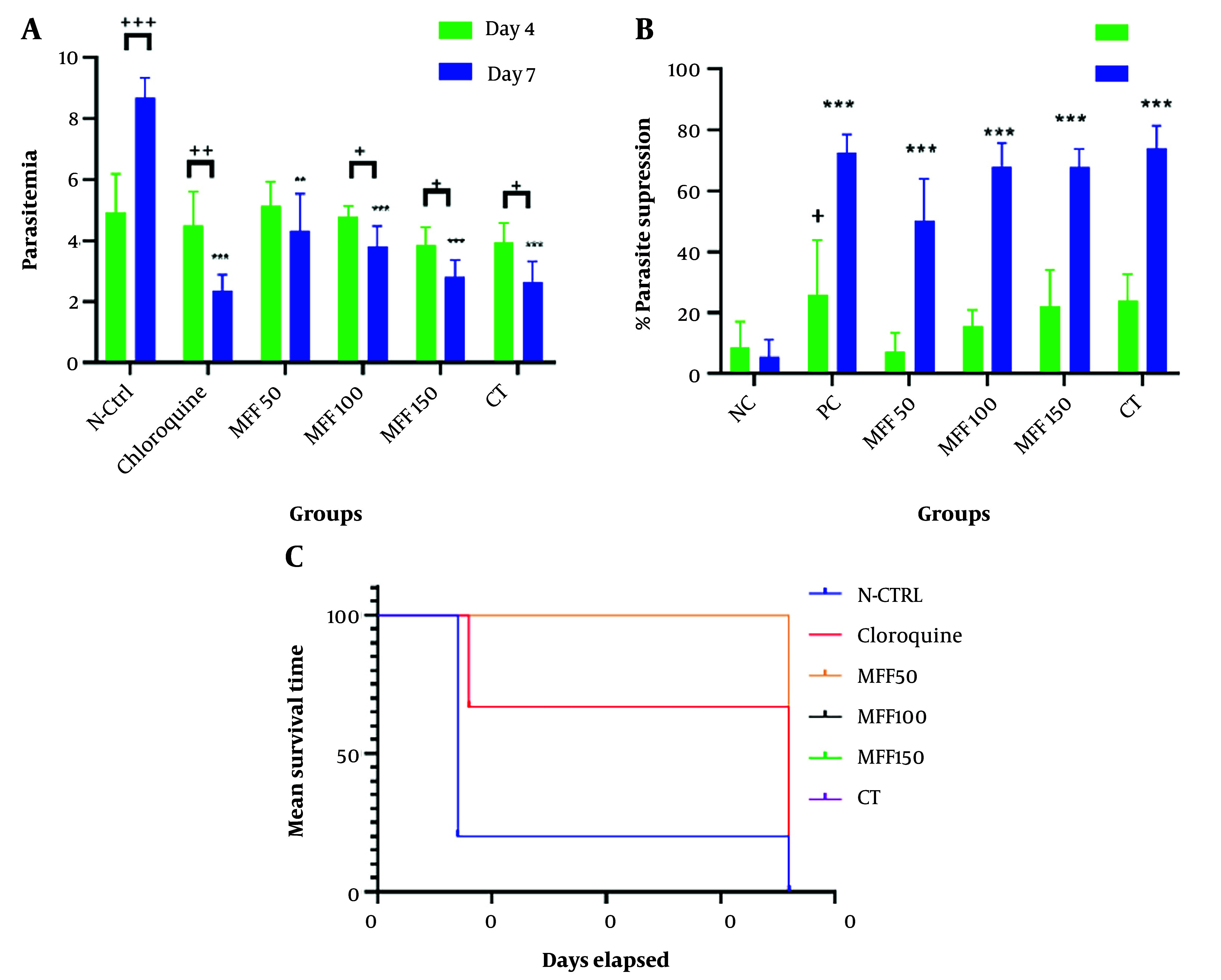
A, mean parasitemia percentages (*, **, ***: P < 0.05, 0.01, 0.001 vs. negative control; +, ++, +++: P < 0.05, 0.01, 0.001 between days 4 - 7); B, parasite inhibitory effects of methoxylated flavonoid fraction (MFF) (cirsimaritin-dominant) at 50, 100, and 150 mg/kg, CT (MFF + chloroquine, 100 + 10 mg/kg), NC (vehicle), and chloroquine (10 mg/kg, PC) on days 4 and 7 (+: P < 0.05 vs. vehicle on day 4; *, **, ***: P < 0.05, 0.01, 0.001 vs. vehicle on day 7); and C, mean survival times under the same treatments over 36 days.

Compared with our previous work on the semipolar extract (SPE) of *A. kopetdaghensis* (7), the MFF displayed a distinct antimalarial profile. Parasitemia levels declined in all treatment groups, with MFF showing a clear dose-dependent effect. Although the maximal suppression achieved by MFF (around 72% at 150 mg/kg) was lower than that reported for SPE (> 85%), the fraction still demonstrated meaningful suppression of parasite growth. The combination of MFF with chloroquine produced only a modest improvement compared with MFF alone, whereas SPE plus chloroquine previously yielded the strongest synergistic effect (Figure 2A and B).

In terms of survival outcomes, while SPE extended survival variably across doses, MFF treatment resulted in complete protection, with all animals surviving the 36-day observation period. This contrasts with chloroquine alone (positive control), which prolonged survival but did not prevent mortality, and with the negative control, which showed a rapid decline. Thus, despite lower parasitemia suppression, MFF provided more consistent long-term protection (Figure 2C). Overall, these findings suggest that the flavonoid fraction contributes primarily through stabilizing host survival and modulating immune responses, whereas the broader SPE — containing both flavonoids and sesquiterpene lactones — exerts stronger direct antiparasitic activity. The present study, therefore, complements our earlier work by clarifying the specific role of methoxylated flavonoids in antimalarial efficacy.

### 4.3. Immunological Responses

Evaluation of cytokine profiles revealed significant differences between treatment groups, with the Kruskal-Wallis test confirming statistical significance (P < 0.0001). In comparison with our earlier study on the SPE of *A. kopetdaghensis* (7), the MFF produced a distinct and more stable immunological pattern. Both chloroquine and MFF at 100 mg/kg induced marked elevations of IFN-γ by day 4, consistent with activation of a Th1 response. Importantly, by day 7, IFN-γ levels remained higher in the MFF group than in the chloroquine group, suggesting a more sustained pro-inflammatory effect.

**Figure 3.**
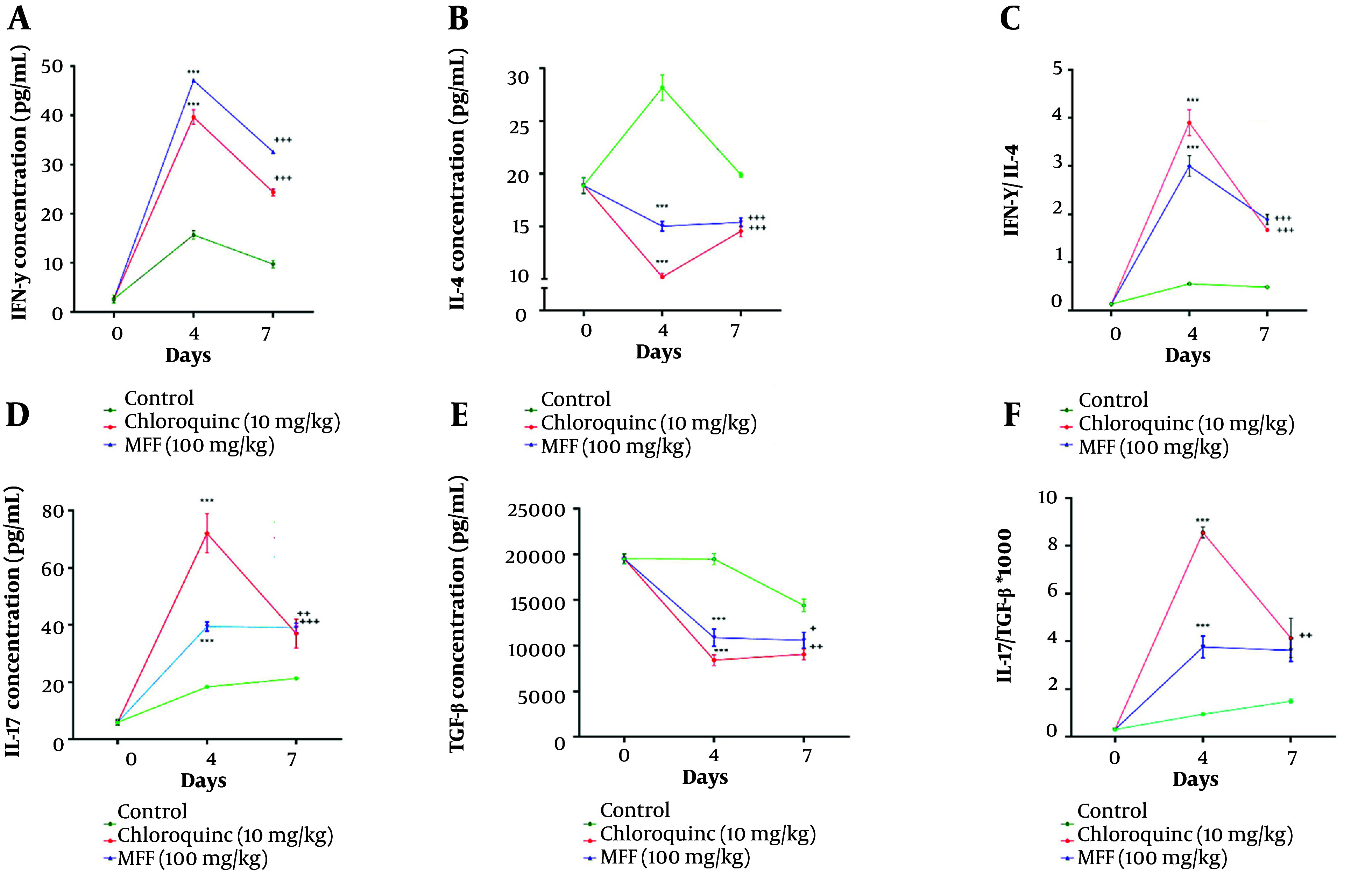
Cytokine responses in *Plasmodium berghei*-infected mice treated with the methoxylated flavonoid fraction (MFF) (cirsimaritin-dominant, 100 mg/kg), chloroquine (10 mg/kg, positive control), or vehicle (placebo). A, interferon-gamma (IFN-γ) levels; B, interleukin-4 (IL-4) levels; C, IFN-γ/IL-4 ratio; D, IL-17 levels; E, TGF-β levels; and F, IL-17/TGF-β ratio, measured on days 0, 4, and 7 post-infection. Statistical significance is indicated as, ***: P < 0.001 vs. control on day 4; +, ++, +++: P < 0.05, 0.01, 0.001 vs. control on day 7.

In parallel, IL-4 levels were suppressed in both groups on day 4; however, by day 7, chloroquine showed weaker suppression, whereas MFF maintained a more balanced reduction. This resulted in a consistently elevated IFN-γ/IL-4 ratio in the MFF group across both time points, indicating a stable shift toward Th1 dominance. Taken together, these findings highlight that, while SPE previously demonstrated broader cytokine fluctuations, MFF provided more controlled modulation of immune responses, fine-tuning the Th1/Th2 balance to maintain effective antiparasitic immunity while limiting excessive Th2 activity (Figure 3A-C).

A similar distinction was observed in the IL-17/TGF-β axis. On day 4, chloroquine elicited the highest IL-17 levels, reflecting a strong early pro-inflammatory response, whereas by day 7, the MFF group at 100 mg/kg surpassed chloroquine, indicating a more sustained activation of the Th17 pathway. In contrast, TGF-β levels were lowest in the chloroquine group at both time points, while MFF maintained a moderate reduction, suggesting better preservation of regulatory activity. The IL-17/TGF-β ratio further emphasized these differences: Chloroquine produced the highest ratio on day 4, reflecting a sharp pro-inflammatory bias, but by day 7, both chloroquine and MFF showed elevated ratios compared with the controls. This indicates that MFF, unlike SPE, promotes more balanced immune modulation, sustaining IL-17 activity while preventing excessive suppression of TGF-β. Such regulation may contribute to the improved survival outcomes observed in MFF-treated mice and underscores the specific role of methoxylated flavonoids in fine-tuning host immunity during malaria infection (Figure 3D-F).

### 4.4. Receptor-Ligand Interactions by in silico Studies

Docking and molecular binding analyses were performed to investigate the interactions between cirsimaritin, the dominant constituent of MFF, and IFN-γ, TGF-β, IL-4, and IL-17 receptors. The binding results obtained for these interactions are presented in Table 1. Docking algorithms were used to evaluate molecular interactions between receptors and compounds to determine the most stable conformations, characterized by the lowest energy states. Negative binding energy indicates a greater binding affinity of ligands to the receptors, including IFN-γ, TGF-β, IL-4, and IL-17.

**Table 1.** Binding Energy (kcal/mol) of Cirsimaritin and Chloroquine with Cytokine Receptors

Variables	Receptor Binding Energies (Kcal/mol)			
	IFN-γ	TGF-β	IL-4	IL-17
**Cirsimaritin**	-7.99	-7.29	-9.11	-7.54
**Chloroquine**	-7.23	-6.96	-8.65	-7.14

Abbreviations: IFN-γ, interferon-gamma; IL-4, interleukin-4.

**Figure 4.**
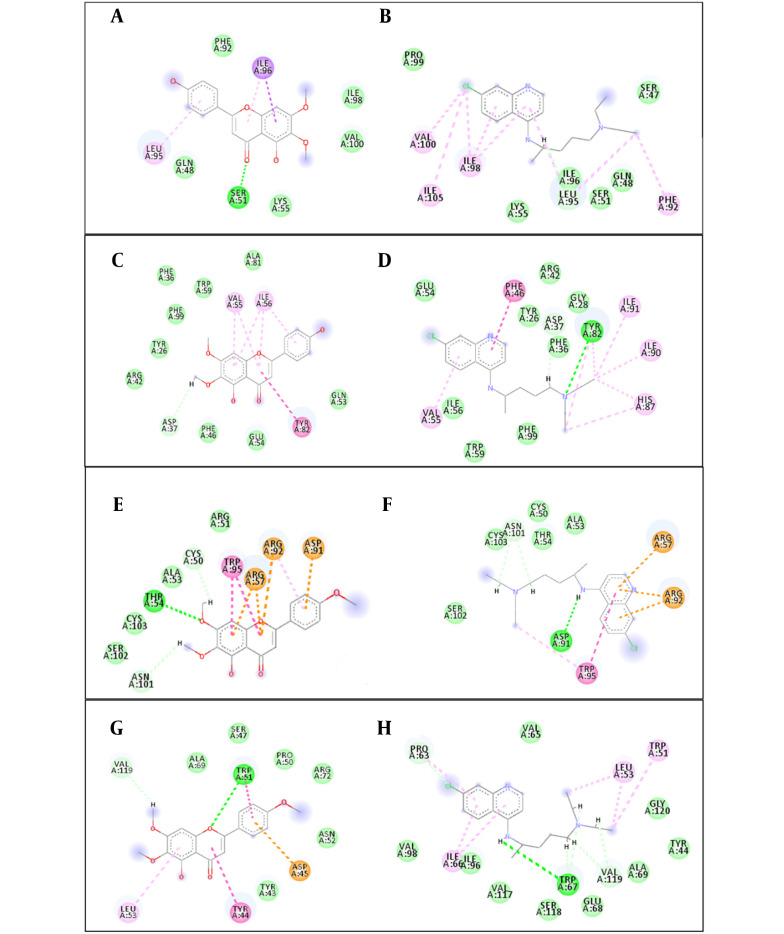
2D interactions of cirsimaritin with interferon-gamma (IFN-γ), TGF-β, interleukin-4 (IL-4), and IL-17 compared with chloroquine. A, cirsimaritin–IFN-γ; B, chloroquine–IFN-γ; C, cirsimaritin–TGF-β; D, chloroquine–TGF-β; E, cirsimaritin–IL-4; F, chloroquine–IL-4; G, cirsimaritin–IL-17; and H, chloroquine–IL-17.

The docking analysis revealed that the IFN-γ/cirsimaritin complex achieved a docking score of -7.99 kcal/mol. The interactions between IFN-γ and cirsimaritin and chloroquine compounds were classified into five categories based on Figure 4A and B: π-alkyl, van der Waals, π-sigma, carbon–hydrogen bonds, and conventional hydrogen bonds. Specifically, residues Phe92, Ile98, Val100, Gln48, and Lys55 exhibited van der Waals interactions, while a π-sigma interaction was observed with residue Ile96. In addition, Leu95 formed a π-alkyl bond with cirsimaritin.

The binding score for the cirsimaritin/TGF-β complex was -7.29 kcal/mol. As shown in Figure 4C and D, the interactions between TGF-β and cirsimaritin and chloroquine compounds were categorized into π-alkyl, carbon–hydrogen bonding, π-π stacking, van der Waals, and conventional hydrogen bonds. Residues Gln53, Glu54, Phe46, Arg42, Tyr26, Phe99, Phe36, Trp59, and Ala81 showed van der Waals interactions. In addition, one π–π stacking interaction and two π-alkyl interactions were identified with Tyr82 and Ile56 residues, respectively.

The binding score for the cirsimaritin/IL-4 complex was -9.11 kcal/mol. Figure 4E and F categorize the interactions between IL-4 and cirsimaritin/chloroquine into seven classes: Van der Waals interactions, conventional hydrogen bonds, carbon–hydrogen interactions, π-cation interactions, π-anion interactions, π–π stacking interactions, and π-alkyl interactions. Conventional hydrogen bonds were observed with Thr54, while Trp95 and Asp91 were involved in π-π stacking and π-anion interactions, respectively.

Finally, the cirsimaritin/IL-17 complex showed a docking score of -7.54 kcal/mol. As shown in Figure 4G and H, the interactions between IL-17 and cirsimaritin/chloroquine were grouped into six classes: π-π stacking, π-alkyl, π-anion, van der Waals, carbon–hydrogen bonding, and conventional hydrogen bonds. Van der Waals interactions were observed for residues Ala69, Ser47, Pro50, Arg72, Asn52, and Tyr43. A π-π stacking interaction, a π-alkyl interaction, and a π-anion interaction were observed with Tyr44, Leu53, and Asp45 residues, respectively.

## 5. Discussion

This study demonstrates the antimalarial potential of the MFF of *A. kopetdaghens*, highlighting both its immunomodulatory effects and molecular interactions. Unlike our previous work on the SPE, which contained a mixture of flavonoids and sesquiterpene lactones, the present investigation isolates and characterizes specific flavonoids — cirsimaritin, cirsilineol, and 6-methoxytricin — and evaluates their biological activity in vivo and in silico. The identification of these constituents provides mechanistic clarity regarding the role of flavonoids in parasite suppression and immune regulation.

In vivo, MFF reduced parasitemia in a dose-dependent manner and prolonged survival, but its most distinctive feature was the stability of immune modulation. The fraction sustained IFN-γ and IL-17 responses while maintaining balanced suppression of IL-4 and TGF-β, thereby fine-tuning the Th1/Th2 and Th17/Treg axes. This contrasts with the broader cytokine fluctuations observed in our earlier SPE study (2).

Although the present study is a mechanistic follow-up, SPE contains a mixture of different compounds, mostly terpenoids such as sesquiterpene lactones, whereas the current work focuses specifically on the MFF to clarify the role of flavonoids in antimalarial activity. A direct side-by-side comparison of SPE and MFF under identical conditions was not performed, which is a limitation. Future studies should test isolated sesquiterpene lactones and flavonoids together in the same experiment to confirm their distinct pharmacological roles.

Such immunological stability may explain the consistent survival outcomes in MFF-treated mice, even when parasite suppression was less pronounced than with the total extract. These findings align with and extend observations from other antimalarial studies. Onjaijan and Somsak reported that allicin and artesunate significantly reduced parasitemia in *P. berghei*-infected mice, with allicin acting through antioxidant and immune-modulating mechanisms—an effect comparable to the immunoregulatory role observed here for MFF (15). Similarly, Ashraf et al. demonstrated that *Artemisia* species exert antimalarial activity independent of artemisinin, highlighting the contribution of secondary metabolites such as flavonoids, consistent with our identification of cirsimaritin as a dominant active compound (16). Angulo and Fresno emphasized the importance of Th1/IFN-γ activation followed by Th2 modulation to prevent tissue damage, a dynamic also observed in our cytokine analyses, where MFF sustained IFN-γ early and balanced IL-4 later (17). However, beyond immunomodulatory effects, Ferreira et al. demonstrated that flavonoids combined with artemisinin act partly through metal ion chelation, a mechanism that may also contribute to the effects of MFF (2).

In the docking study on cytokine protein targets, this research confirmed that specific amino acids play a vital role in the binding interactions of IFN-γ, IL-4, TGF-β, and IL-17 with their respective ligands. This study identified Phe92, Ile98, Val100, Gln48, Lys55, and Ile96 as critical residues involved in van der Waals and π-sigma interactions with cirsimaritin as a ligand. These findings are consistent with another study that emphasized the importance of Val100 and Gln48 in the catalytic mechanism of IFN-γ (18). In addition, the involvement of residues Ser51 and Ser47 in hydrogen bonding supports the critical role of such interactions in stabilizing the IFN-γ–ligand complex (19).

Similarly, residues Gln53, Glu54, Phe46, Arg42, Tyr26, Phe99, Phe36, Trp59, and Ala81 were found to be involved in van der Waals interactions with cirsimaritin/chloroquine. Previous research has shown that Arg42 and Tyr26 are essential for the catalytic activity of TGF-β, supporting our findings regarding their involvement in ligand interactions (20). Furthermore, our results show that Lys88 and Thr54 are crucial for hydrogen bonding between IL-4/IL-17 and cirsimaritin/chloroquine. These interactions are consistent with other reports highlighting the role of Thr and Lys residues in stabilizing the IL-4–ligand complex through hydrogen bonds, confirming our binding predictions (21).

The spontaneous formation of four complexes (IFN-γ/cirsimaritin, TGF-β/cirsimaritin, IL-4/cirsimaritin, and IL-17/cirsimaritin) is mainly driven by hydrogen bonds and van der Waals forces, reflecting the non-covalent nature of these interactions. This enhances the understanding of receptor–ligand interactions and demonstrates that non-covalent forces, such as van der Waals interactions, hydrogen bonding, and π–π stacking, contribute to binding affinity and specificity (22). Identifying these key residues provides valuable guidance for designing more potent and selective ligands. Targeting these residues may enhance affinity and specificity for new ligand candidates. Additionally, understanding the role of non-covalent interactions in stabilizing receptor–ligand complexes can aid in designing ligands with improved pharmacokinetic properties (23).

Although cirsimaritin is the major constituent of MFF, the fraction also contains cirsilineol and 6-methoxytricin. Molecular docking was performed only for cirsimaritin as a representative compound to support the in vivo findings. Docking of the other two flavonoids would provide a more comprehensive mechanistic explanation and should be addressed in future studies.

### 5.1. Conclusions

Through preparative HPLC and detailed NMR analysis, we successfully isolated and identified the MFF of *A. kopetdaghensis*, composed of cirsimaritin (dominant), cirsilineol, and 6-methoxytricin. Unlike our previous study on the SPE, which examined a broader mixture of plant constituents, the present work focuses specifically on flavonoids as discrete bioactive agents.

In vivo experiments demonstrated that MFF, administered alone or in combination with chloroquine, significantly prolonged survival in *P. berghei*-infected mice, reduced parasitemia, and modulated host immune responses. Importantly, molecular docking analyses provided mechanistic confirmation of these findings, revealing strong binding affinities of cirsimaritin to cytokine receptors (IFN-γ, IL-4, IL-17, and TGF-β). These receptor-level interactions explain the observed balance between pro-inflammatory and regulatory pathways, underscoring the role of flavonoids in fine-tuning the Th1/Th2 and Th17/Treg axes during malaria infection.

Taken together, this study extends our earlier work by moving from extract-level observations to constituent-specific insights. By combining the isolation and structural identification of flavonoids with in silico docking confirmation, we demonstrate that methoxylated flavonoids, particularly cirsimaritin, are key modulators of host immunity and promising candidates for further development as adjuncts or leads in antimalarial therapy.

## Data Availability

The spectral data presented in this study were uploaded during submission as a supplementary file and are openly available to readers upon request.
